# Therapeutic potential and possible mechanisms of ginseng for depression associated with COVID-19

**DOI:** 10.1007/s10787-023-01380-0

**Published:** 2023-11-27

**Authors:** Fangyi Zhao, Kai Zhang, Hongyu Chen, Tianqi Zhang, Jiayu Zhao, Qianyu Lv, Qin Yu, Mengyu Ruan, Ranji Cui, Bingjin Li

**Affiliations:** 1 Jilin Provincial Key Laboratory on Molecular and Chemical Genetics, The Second Hospital of Jilin University, Changchun, People’s Republic of China; 2 Engineering Laboratory for Screening of Antidepressant Drugs, Jilin Province Development and Reform Commission, Changchun, People’s Republic of China; 3 Jilin Provincial Key Laboratory on Target of Traditional Chinese Medicine with Anti-Depressive Effect, Changchun, People’s Republic of China

**Keywords:** Ginseng, COVID-19, Depression, Cytokine storm, ACE2 receptor

## Abstract

Recently, a global outbreak of COVID-19 has rapidly spread to various national regions. As the number of COVID-19 patients has increased, some of those infected with SARS-CoV-2 have developed a variety of psychiatric symptoms, including depression, cognitive impairment, and fatigue. A distinct storm of inflammatory factors that contribute to the initial disease but also a persistent post-acute phase syndrome has been reported in patients with COVID-19. Neuropsychological symptoms including depression, cognitive impairment, and fatigue are closely related to circulating and local (brain) inflammatory factors. Natural products are currently being examined for their ability to treat numerous complications caused by COVID-19. Among them, ginseng has anti-inflammatory, immune system stimulating, neuroendocrine modulating, and other effects, which may help improve psychiatric symptoms. This review summarizes the basic mechanisms of COVID-19 pneumonia, psychiatric symptoms following coronavirus infections, effects of ginseng on depression, restlessness, and other psychiatric symptoms associated with post-COVID syn-dromes, as well as possible mechanisms underlying these effects.

## Introduction

2019 Coronavirus disease (COVID-19), an illness related to infection with Severe Acute Respiratory Syndrome Coronavirus 2 (SARS-CoV-2), has been an epidemic since it first appeared in 2019 in the Wuhan region of China, then spread rapidly worldwide. It is the twenty-first century's No.3 highly infectious human disease, which has already caused enormous morbidity and mortality worldwide(Asselah et al. 2021; Das et al. 2021). As of 16 August 2023, 769,774,646 definite diseases of COVID-19, inclusive of 6,955,141 deaths, have been reported globally to the World Health Organization (WHO) (https://covid19.who.int/). WHO classified COVID-19 as a global public health emergency in 2019 (Hu et al. 2021; Muralidar et al. 2020; Sharma et al. 2021). COVID-19 is an RNA virus with a higher mutation rate compared to DNA viruses. From the beginning of the epidemic until now, numerous variants of concern (VOCs) including Alpha ((B.1.1.7)), Beta (B.1.351), Gamma (P.1), Delta (B.1.617.2), and Omicron (B.1.1.529) of SARS-CoV-2 have been identified (WHO update), as the virus has quickly evolved. Many patients with pneumonia caused by SARS-CoV-2 are reported to experience persistent psychiatric symptoms such as depression, cognitive impairment, and fatigue during or after the acute phase, which severely affects health and quality of life for a longer period after the initial infection (Tabacof et al. 2022; Sykes et al. 2021; Ceban et al. 2022). Inflammatory responses in the body may contribute to psychiatric symptoms, including suicidal ideation in the patient population (Sher 2021). The frequency of persistent psychiatric symptoms in patients after mild SARS-CoV-2 pneumonia ranges from 10 to 35% (van Kessel et al. 2022) (Xiong et al. 2021). This may be directly related to the effect of the virus on the brain, as coronaviruses could access the brain through the neuro-mucosal interface that crosses the olfactory mucosa (Meinhardt et al. 2021). There are two mechanisms involved in SARS-CoV-2 infections. One of these involves angiotensin-converting enzyme 2 (ACE2) receptors that are situated on the cell surface and are widely distributed in the human respiratory and digestive tracts (Scialo et al. 2020). The other mechanism is related to cytokine function. There are multiple inflammatory factors in the body, including interleukin-10 (IL-10), interleukin-6 (IL-6), macrophage chemoattractant protein-1 (MCP-1), and tumor necrosis factor-α (TNF-α) (Lu et al. 2021). It has been reported that central and peripheral nervous system complications from COVID-19 may be associated with a cytokine storm caused by SARS-CoV-2 (Thepmankorn et al. 2021). ACE2 receptors and inflammation are also closely related to psychiatric symptoms observed after SARS-CoV-2 infection. As such, drugs that modulate the levels of ACE2 receptors or cytokines following viral infection are one way to prevent or treat post-coronavirus syndrome (Tsai et al. 2021). Ginseng is a traditional Chinese medicine, widely used in Asian countries including Korea, Japan, and China (Lin et al. 2016) (Kiefer and Pantuso 2003) (Choi 2008) (Zhang et al. 2020). In China, ginseng is the "king of herbs" and "the first of the three treasures of traditional chinese medicine". Ginseng was reported to have pharmacological effects on anti-inflammatory, antioxidant, immunostimulant, and antiaging effects(Liu et al. 2021a; Shin and Cho 2023; Han et al. 2022; Kwon et al. 2023; Su et al. 2023). In addition, ginseng can regulate the excitability of neurons in the central nervous system (CNS) and produce neuroprotective effects (Liu et al. 2020a; Gong et al. 2022; Kim et al. 2022). According to the different preparation methods, ginseng can be divided into different types, such as raw ginseng, white ginseng, red ginseng, and black ginseng (Jin et al. 2015; Huang et al. 2023). Ginseng has been identified as containing numerous biologically active ingredients, such as ginsenosides, polysaccharides, phenolic compounds, and proteins (Kang et al. 2007; Liu et al. 2021b). The primary active ingredient in ginseng is ginsenoside, which is divided into three main chemical types, including oleic acid, protopanaxadiol (PPD), and protopanaxatriol (PPT) types. Ginsenosides may regulate virus-induced tissue damage, local or systemic inflammation, and immune function, thereby reducing the cardiac burden, finally protecting the CNS (Liu et al. 2021b; Wang et al. 2023; Yi 2022). The above actions are linked to treatments for post-COVID-19 symptoms. Studies have shown that ginseng produces antidepressant-like effects mainly via regulationg inflammatory factors and pathways in several animal models of depression (Li et al. 2022; Kang et al. 2017; Guo et al. 2021). Therefore, this review aims to explore the possibility of ginseng for treating and preventing psychoneurological complications associated with COVID-19.

## The basic mechanisms of COVID-19 pneumonia

Although most patients with COVID-19 pneumonia present with mild symptoms, a minority of patients develop multi-organ dysfunction that may be life-threatening. There are currently two main mechanisms known to underlie this disease: ACE2 receptor activation and inflammation.

### ACE2 (angiotensin-converting enzyme 2) and SARS-CoV

ACE2 is a functional receptor on the surface of somatic cells and is widely expressed in the esophagus, heart, kidney, stomach, lung, and brain. Abnormal ACE2 function produces symptoms that include dry cough, headache, nausea, and diarrhea (Beyerstedt et al. 2021). ACE2 is essentially a component of the renin–angiotensin–aldosterone system (RAAS). RAAS is an important pathway that regulates fluid and electrolyte balance, as well as systemic vascular resistance (Iwai and Horiuchi 2009; Fountain et al. 2023). RAAS includes three vital compounds: renin, angiotensin II, and aldosterone (Fountain et al. 2023). A recent study pointed out that SARS-CoV-2 depletes ACE-2, leading to a skewed activation of RAAS, reduced blood perfusion, and promotion of inflammation (Miners et al. 2020). Viral entry into host cells affects ACE/ACE2 function and disturbs RAAS homeostasis, leading to progressive COVID-19 disease (Beyerstedt et al. 2021) (Leung and Sin 2020) (Faheem et al. 2020). SARS-CoV-2 competes with Ang II in vivo for ACE2, which is the target of SARS-CoV-2 to enter cells. SARS-CoV-2 targets ACE2, over-activates ACE2, decreases ACE expression in membranes, and promotes RAAS imbalance (Glowacka et al. 2010). RAAS is composed of two primary pathways: the ACE-angiotensin II (Ang II)-Ang II receptors AT1/AT2 pathway and the ACE2-angiotensin (1–9)-angiotensin (1–7)-Mas pathway. The balance between these two systems is an important part of maintaining RAAS homeostatic balance. Extreme and prolonged imbalance in either direction produces pathological effects. The Ang II-Ang II receptors AT1/AT2 pathway stimulates multiple biological functions including vasoconstriction, cell proliferation, and inflammation. By contrast, the ACE2-angiotensin (1–9)-angiotensin (1–7)-Mas pathway mediates the opposite biological functions including vasodilation, anti-proliferation, and anti-inflammation. It has been noted that elevated plasma ANG II and aldosterone levels positively correlate with COVID-19 severity (Wu et al. 2020). Coronaviruses have four key structural proteins: envelope (E), membrane (M), nucleocapsid (N), and spike (S) proteins. ACE2 binds with the S protein of SARS-CoV2 and thereby enters the cell by endocytosis. The structural domain binding to ACE2 in the host cell is required for initiation of the S protein activation along with the transmembrane protease serine protease-2 and a disintegrin and metallo2 proteinase metallopeptidase domain 17 (ADAM17) (Xu et al. 2020). These two pathways also have a role in the central nervous system CNS. The former decreases neuronal survival and is associated with anxiety and depression, while the latter increases neuronal survival, and decreases anxiety and depression (de Melo and Almeida-Santos 2020). Therefore, targeting ACE2 is considered one of the promising potential approaches to the treatment of coronavirus-associated psychiatric symptoms including depression.

### Inflammatory cytokines and SARS-CoV-2

**Fig. 1:**
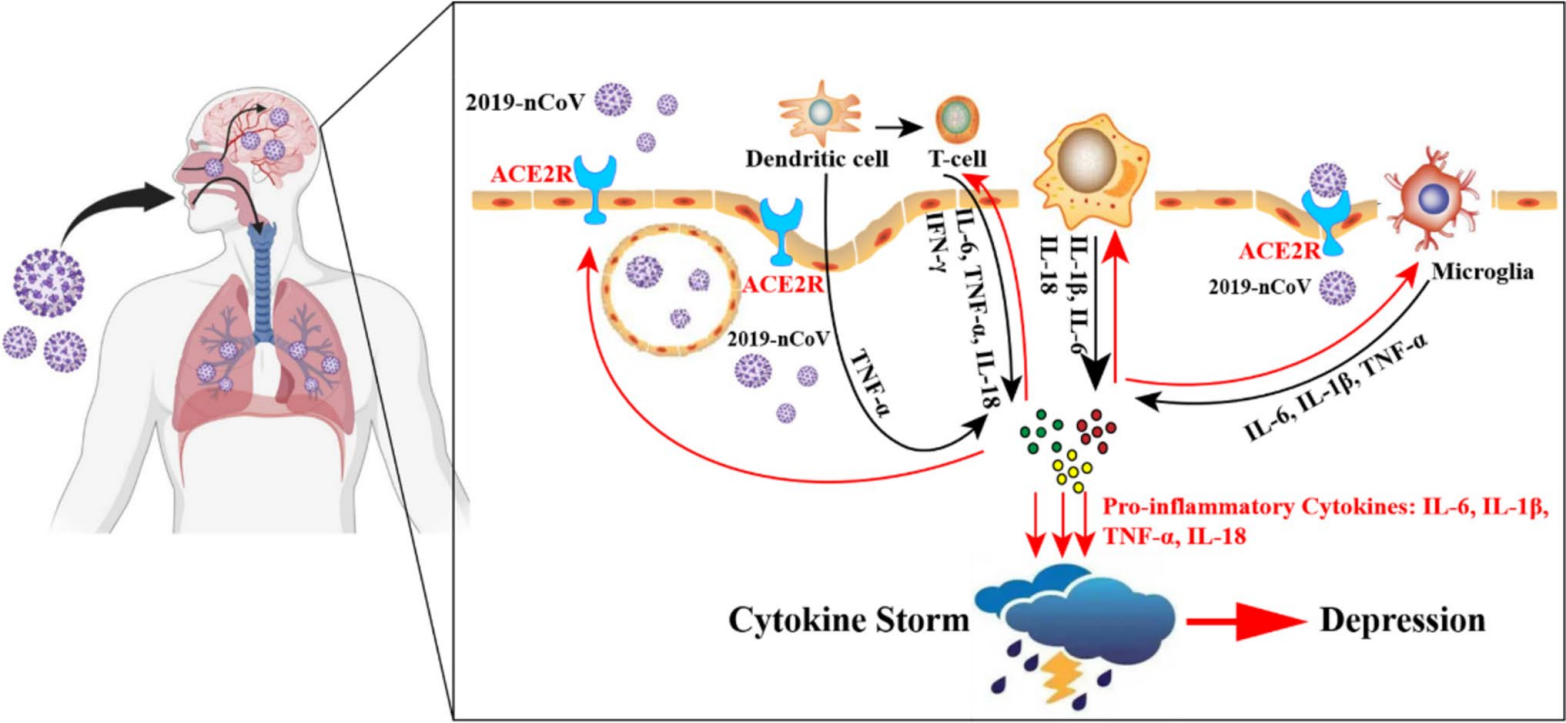
The SARS-CoV2 infection initiates the activation and aggregation of multiple immune cells, including dendritic cells, macrophages, and lymphocytes, resulting in a strong inflammatory response triggered by the release of pro-inflammatory cytokines (Jiang et al. 2020a). *Note*: ACE2R, Angioten-sin-converting enzyme 2 receptors; IL-6, Interleukin-6; TNF-α, tumor necrosis factor-α; IL-18, In-terleukin-18; IFN-γ, Interferon-gamma; IL-1β, Interleukin-1 beta

As shown in Fig. 1, the SARS-CoV2 infection triggers the activation and aggregation of a variety of immune cells including dendritic cells, macrophages, lymphocytes (T cells, B cells, natural killer (NK) cells, and gamma-delta T (gd T) cells), leading to strong inflammatory responses triggered by the release of pro-inflammatory cytokines and chemokines (C-base sequence chemokine ligands CCL, such as CCL-2, CCL-3 and CCL-10). Macrophages produce IL-1β, IL-6 and IL-8. T-cells secrete IFN-γ, IL-6, IL-18 and TNF-α. Dendritic cells secrete TNF and also activate T-cells. IL-6 was also secreted by Fibroblasts. (Sun et al. 2020). Microglia also secrete IL-1β, TNF-α, and IL6, which could push other immune cells in a positive feedback loop. It has been reported that significant neuroinflammation in the brainstem was found in autopsies of patients with COVID-19, which involved microglia aggregation (Schwabenland et al. 2021). Cytokines are messenger molecules of the immune system, including IL-6, IFN-α, IL-1β, IL-8, IL-10, IFN-γ, TNF-a, and C-reactive protein (CRP), and as such, they are closely related to the worst outcomes of COVID-19 (McElvaney et al. 2020; Khaksarinejad et al. 2022). Cytokine storms are critical in the infection process. The prognosis is significantly worsened by the overproduction of pro-inflammatory factors, which preferentially target lung tissue (Yokota et al. 2021) (Post et al. 2020). Cytokine storm not only eliminates pathogenic microorganisms but also causes host tissue damage affecting a wide variety of organs including the brain (Tang et al. 2020), contributing to the death of SARS-CoV-2 infected patients (Qin et al. 2020). Recent findings have shown that SARS-CoV-2 viral proteins in the nasal cavity result in significant activation of microglia in the olfactory bulb, thereby affecting brain function (Käufer et al. 2022). Besides, inflammasomes are intracellular protein signaling complexes that play an important role in the activation of inflammatory responses (Zheng et al. 2020). Inflammasomes are mainly divided into two classes canonical and non-canonical inflammasomes. Nucleotide-binding and oligomerization domain (NOD)-like receptor NLRP3 is one of the canonical inflammasomes, react with SARS-CoV-2 spike protein or ACE2 inducing hyper-inflammation and over-producing pro-inflammatory cytokines (Olajide et al. 2021; Tefferi et al. 2021; Li et al. 2003; Kuba et al. 2005; Ratajczak et al. 2021). Cytokine storm is also increased due to the internalization of ACE2 receptors in SARS-CoV-2 infected cells. Infection with SARS-CoV-2 reduced cell surface ACE2 levels and increased the relative levels of angiotensin II activity, which binds to AT1R and leads to vasoconstriction, increased ROS levels, and NF-kB-mediated inflammation (Vellingiri et al. 2020). COVID-19 can cause severe encephalitis and neurological disorders, including stroke, neurovascular unit damage, and blood–brain barrier disruption.

## Possible common mechanisms of COVID-19 and depression

It has been shown that depression is the most frequently observed psychiatric sequela after the SARS-CoV-2 infections (Mazza et al. 2020, 2021; Chen et al. 2020, Renaud-Charest et al. 2021). Meta-analysis showed that 45% of COVID-19 patients with depression(Deng et al. 2021). The pathogenesis of depression mainly includes the monoamine neurotransmitter hypothesis, neurotrophic factor hypothesis, neuroendocrine hypothesis, and neuroplasticity hypothesis (Milaneschi et al. 2019; Gelle et al. 2021; Zajkowska et al. 2022; Price and Duman 2020; Beurel et al. 2020). In addition, several reports support a link between depression and inflammatory processes, and the connection appears to be bidirectional (Beurel et al. 2020; Kohler et al. 2016; Leonard 2018; Liu et al. 2019). The inflammatory hypothesis of depression indicates that depression is, in part, the result of changed immune-inflammatory homeostasis (Berk et al. 2013). Studies have demonstrated that patients with MDD (major depressive disorder) exhibit increased peripheral blood inflammatory biomarkers, such as IL-6, TNF-α, IL-10, C-C motive chemokine ligand 2(CCL2), IL-13, IL-18, IL-12, and CRP (Liu et al. 2020b; Köhler et al. 2017; Perry et al. 2021; Felger et al. 2020). It has been reported that various antidepressants including sertraline, decrease IL-6, IL-2, IL-12, and TNT-α (Jeon and Kim 2016). MDD and COVID-19 were both correlated with levels of activated microglia (Dowlati et al. 2010) (Köhler et al. 2017) and microglia also promote neuroinflammation (Yirmiya et al. 2015). Inflammatory cytokines have been shown to access the brain and interact with almost all pathophysiological mechanisms known to be associated with depression, including neurotransmitter metabolism, neuroendocrine function, and neuroplasticity (Miller et al. 2009).

Furthermore, an increase in levels of NLRP3 correlated with depressive-like behaviors in lipopolysaccharide (LPS), restraint stress, and ovariectomized (OVX)-induced depression models (Xia et al. 2023; Iwata et al. 2016; Alcocer-Gómez et al. 2014, 2017). Activated NLRP3 inflammasome has also been found in MDD patients, which may be associated with high levels of blood IL-1β and IL-18(Alcocer-Gómez et al. 2014; Syed et al. 2018). Apart from Inflammatory cytokines, ACE2 is also involved in pathophysiology of the depression. An epigenetic study showed that serum methylation of the regulatory region of the *ACE2* gene is changed in depressed patients (Zill et al. 2012). Antidepressant efficacy is affected by *ACE2* polymorphisms (Bahramali et al. 2016; Baghai et al. 2004). For example, the *ACE2* genetic variant G8790A has been correlated with differential responses to selective 5-hydroxytryptamine reuptake inhibitors (SSRIs), such as sertraline (Firouzabadi et al. 2022). Evidence from preclinical models also suggests a role for these systems in depression and antidepressant responses. the ACE inhibitor captopril (30 mg/kg, i.p.) decreased immobility time in the forced swimming test in mice and learned helplessness in that model(Giardina and Ebert 1989; Martin et al. 1990). ACE inhibitors (enalapril and ramipril) produce antidepressant-like effects in STZ-induced diabetes-associated depression by regulating hippocampal BDNF (Balogh et al. 2020). Overexpression of Ang-(1–7) or ACE2 produces anxiolytic effects in the transgenic rat models, and Mas signaling is involved in this phenomenon (Kangussu et al. 2017; Wang et al. 2016a).

## Effects of ginseng on COVID-19 pandemic caused depression

### Ginseng alleviates COVID-19 pandemic-caused depression by affecting ACE receptors

The main active component of ginseng is ginsenoside. Until now more than 150 structurally similar ginsenosides have been isolated from ginseng. Ginsenosides are generally classified into two groups based on their chemical structure: four-ring dammarane type and five-ring oleanane type. Dammarane type of the ginsenoside include ginsenosides Rb1, Rd, Rb2, Re, and Rg1, they are also the most abundant ginsenosides in ginseng (Jin et al. 2022)*.* Oleanane-type ginsenosides, such as Ro, are not usually detectable in *P. ginseng* (Kim et al. 2018). Ginseng may also affect ACE function. Clinical trials in recent years have found that ginsenosides can alleviate hypertension and improve vascular activity and a variety of other risk factors for cardiovascular disease. Ginseng extract inhibits ACE (Ali et al. 2021). Using molecular docking analysis to model the interaction of these molecules with ACE, ginsenosides were found to inhibit ACE by hydrogen binding and hydrophobic interaction with the catalytic residues of the C- and N- structural domains of ACE and zinc ions, thereby blocking the catalytic activity of ACE (Ali et al. 2021). Another study found that ginseng extract G115 had a significant inhibitory effect on ACE activity in human endothelial cells but did not affect the production of NO (Persson et al. 2006). Whether ACE is involved in the antidepressant-like effects of ginseng, and other immune mechanisms that may underlie such effects, requires further study.

### Ginseng alleviates COVID-19 pandemic-caused depression by regulating inflammatory factors

Table 1 shows the possible mechanisms of the inflammation involved in the antidepressant-like effects of ginseng. As shown in Table 1, the active ingredient of ginseng produces an antidepressant-like effect in several animal models of depression, including lipopolysaccharide (LPS), chronic unpredictable mild stress (CUMS), and chronic restraint stress (CRS), by regulating inflammatory factors. The most common mechanisms in these studies find that ginsenosides can inhibit inflammation by reducing the levels of IL-6, IL-1β, TNF-α, and the NF-kB pathway, or by reducing glial cell activation in depressive animal models (Wang et al. 2016b, Jiang et al. 2022b; Zhang et al. 2021). Besides, various components of ginseng such as Ginsenoside 20(S)-protopanaxadiol, Ginsenoside Rh2, Ginsenoside Rk1, Ginsenoside Rd, and Ginsenoside Rb1 have a wide range of biological activities such as antioxidant and anti-inflammatory (Wang et al. 2016b; Zhang et al. 2021; Jiang et al. 2020a, b; Han et al. 2020). The active ingredients of ginseng significantly reduced CORT and pro-inflammatory cytokines (IL-6, IL-1β, and TNF-α) in the serum of the depression model mice as well as reduced levels of iNOS, COX2, caspase3, caspase9, Bax, Bcl-2, NLRP3, and p65 in the hippocampus of the depressive model mice and rats (Xu et al. 2022; Li et al. 2020; Zhan et al. 2022). A study by Choi et al. (2018) found that *P. ginseng* extract (PGE) inhibits HPA axis mechanisms and exerts antidepressant-like effects through anti-neuroinflammatory and antioxidant (nuclear factor erythroid 2 related factor 2/heme oxygenase-1 activation) activities. In addition, PGE increased the expression level of BDNF mRNA and also ameliorated the activation of microglia and neuroinflammation in the amygdala of CRS-induced mice (Choi et al. 2018). Studies have shown that the sesquiterpenoids from the root of *Panax ginseng* (SPG) have significant antidepressant effects, and SPG treatment significantly reduced serum IL-6 and TNF-α levels and increased inhibited superoxide dismutase (SOD) activity in the hippocampus (Wang et al. 2018). Additionally, SPG effectively up-regulated hippocampal BDNF, prothymosin-associated kinase B (TrkB), and sirtuin type 1 (sirt1) expression, and down-regulated the inhibitor of κB-α (IκB-α) and nuclear factor-κB (NF-κB) phosphorylation (Wang et al. 2018). Kang et al. assessed the antidepressant effects of ginseng total saponins (GTS) in an LPS-induced depression-like behavioral model using a variety of depression-related behavioral and biochemical experiments (Kang et al. 2011). It was found that GTS significantly attenuated LPS-induced depression-like behavior. In addition, LPS-induced increases in 5-hydroxytryptamine and tryptophane turnover in the brain were significantly reduced by GTS (Kang et al. 2011). Brain and peripheral indoleamine 2,3-dioxygenase (IDO) activities were also inhibited after pretreatment with GTS (Kang et al. 2011). The recovery from LPS-induced depression-like behavior associated with GTS was accompanied by a decrease in hippocampal mRNA levels of IL-1β, IL-6, TNF-α, and IDO (Kang et al. 2011). Moreover, GTS treatment significantly reduced the production of pro-inflammatory cytokines in LPS-simulated mice and RAW264.7 cells (Kang et al. 2011). Ginsenoside Rg1 is a well-recognized anti-inflammatory agent, and studies have shown that peripheral restriction of Rg1 is effective in attenuating weight loss, anorexic and depressive-like behaviors, and neurochemical disturbances associated with central LPS challenge (Zheng et al. 2014). Biochemical analysis of peripheral neuroimmune mediators suggests that Rg1 attenuates dysregulation of the HPA axis and selectively reduces elevated levels of circulating IL-6 (Zheng et al. 2014). Ginsenoside Rg1 treatment for 3 weeks attenuated depression-related behaviors in chronic unpredictable mild stress (CUMS)-exposed rats, as evidenced by increased sucrose preference, improved locomotor activity, and shortened sedentary time, and these ameliorative effects of ginsenoside Rg1 treatment were associated with modulation of pro-inflammatory cytokine IL-1β (Zhang et al. 2019). Ginsenoside Rg1 reverses CUMS-induced IL-1β elevation, possibly by inhibiting NF-κB pathway activation and modulating the expression of nucleotide-binding oligomerization domain-like receptor family pyrin domain-containing 3 inflammasome expression (Zhang et al. 2019). Fan et al. found that chronic pretreatment with ginsenoside Rg1 prior to stress exposure significantly inhibited inflammatory pathway activity by attenuating the overexpression of pro-inflammatory cytokines and activation of microglia and astrocytes (Fan et al. 2018). In addition, ginsenoside Rg1 inhibited CUMS exposure-induced neuronal apoptosis, increased Bcl-2 expression, and decreased cleaved Caspase-3 and Caspase-9 expression in the ventral medial prefrontal cortex (vmPFC) region, and ginsenoside Rg1 increased the expression of nuclear factor erythroid 2-related factor (Nrf2) expression and inhibit p38 mitogen-activated protein kinase (p-p38 MAPK) and nuclear factor κB (NF-κB) p65 subunit activation within the vmPFC (Fan et al. 2018). Oral administration of ginsenoside Rg1 to rats resulted in a dose-dependent decrease in the production of both NO and TNF-α in rat cerebral cortex and hippocampal tissue(Hu et al. 2011). Ginsenoside Rg1 inhibited microglia activation by suppressing Iba-1 expression (Hu et al. 2011). In addition, ginsenoside Rg1 inhibited the expression of inducible nitric oxide synthase (iNOS), and ginsenoside Rg1 suppressed LPS-induced levels of IκB phosphorylation, nuclear translocation of the p65 subunit of NF-κB, and phosphorylation levels of p38, ERK1/2, and JNK mitogen-activated protein kinase (MAPK) (Hu et al. 2011). This suggests that Rg1 suppresses LPS-mediated inflammation by inhibiting NF-κB and MAPK pathways. Li et al. (2022) showed that ginsenoside Rg1 treatment attenuated depressive-like behavior, microglia activation, and mitochondrial dysfunction in CRS rats. Oral administration of ginseng Rg3 attenuated LPS-induced disturbed hippocampal tryptophan and serotonin turnover in depressive-like mice while decreasing mRNA expression of pro-inflammatory cytokines and indoleamine-2,3-dioxygenase (IDO) and the central benefits were partially related to activation of microglia and regulation of the NF-κB pathway (Kang et al. 2017). In addition, Rg3 significantly reduced LPS-induced elevations of IL-6 and TNF-α in plasma and restored systemic homeostasis in tryptophan-kynurenine metabolism (Kang et al. 2017). Oral doses of ginsenoside Rg3 at 20 and 30 mg/kg significantly attenuated the up-regulation of TNF-α, IL-1β, and IL-6 mRNA in brain tissues 4 h after LPS injection (Park et al. 2012). Ginsenoside Rg3 (30 mg/kg) reduced the morphological activation of microglia by LPS and the expression of the Iba-1 protein (Park et al. 2012). In addition, oral administration of ginsenoside Rg3 (30 mg/kg) reduced the expression of iNOS and cyclooxygenase-2 (COX-2) in brain tissue (Park et al. 2012). Ginsenoside Rb1 (GRb1), a major constituent of ginseng, is known to inhibit the inflammatory cascade and alleviate depressive-like behavior (Zhang et al. 2021; Liang et al. 2022). Studies have shown that oral administration of GRb1 alleviates depressive-like behavior in CRS model mice, which may be due to the reduction of hippocampus protein expression of IL-1β, TNF-α and ionized calcium-binding adapter molecule 1 by increasing the brain-derived neurotrophic factor and phosphorylated protein kinase B/protein kinase B (p-AKT/AKT) protein expression and reducing the serum IL-1β and TNF-α levels (Guo et al. 2021). In addition, Ginsenoside Rb1 decreased the protein expression of IL-1β and TNF-α in LPS-induced BV-2 microglia (Guo et al. 2021; Jiang et al. 2022b). Furthermore, ginsenoside Rb1 administration significantly reduced the protein expression of NLRP3 (inflammasome) as well as facilitated the activation of Nrf2, HO-1, and Sirtuin1 (SIRT1) protein expression in the hippocampus (Jiang et al. 2022b). Liang et al. demonstrated that ginsenoside Rb1 inhibits peripheral and hippocampal inflammation through MAPK/NF-κB signaling. In inflammation-mediated depression, ginsenoside Rb1 ameliorated glucocorticoid receptor damage and 5-HT_1A_ receptor expression (Liang et al. 2022). In addition, ginsenoside Rb1 increases 5-HT levels and inhibits indoleamine 2,3-dioxygenase activity (Liang et al. 2022). Han et al. showed that ginsenoside Rd treatment significantly attenuated stress-induced anxiety/depression-like behaviors and reduced blood corticosterone levels (Han et al. 2020). Treatment of ginsenoside Rd suppressed stress-induced NF-κB activation and NF-κB+/Iba1+ cell populations in the hippocampus, and increased BDNF expression and BDNF+/NeuN+ cell population (Han et al. 2020). Ginsenoside Rh2 is also one of the main active ingredients in ginseng. A study by Wang et al. (2016b) found that mice treated with ginsenoside Rh2 improved depressive-like behavior induced by colorectal carcinoma, which appeared to be achieved by lowering depression-associated cytokines, IL-6, IL-18, and TNF-α. Ginsenoside Rh2, a major bioactive compound with anti-T-cell inflammatory activity extracted from ginseng, attenuates microglia activation through the toll-like receptor 4 (TLR4)/NF-κB signaling pathway (Xu et al. 2022). Xu et al. (2022) showed that ginsenoside Rh2 attenuates microglia overactivation via the HMGB1/TLR4/NF-κB signaling pathway and neuroinflammation, thereby improving depressive-like behavior in mice. In addition, studies have shown that Rk1 exerts antidepressant effects through its antioxidant activity, inhibition of neuroinflammation, and positive regulation of the BDNF-TrkB pathway (Li et al. 2020). 20 (S)-protopanaxadiol (PPD) exhibits a wide range of biological activities including antioxidant, anti-fatigue, and anti-inflammatory properties (Jiang et al. 2020a, b).

**Table 1:** Inflammation-mediated antidepressant-like effects of ginseng in animal models

No.	Original sources	Compounds	Depression model	Model preparation	Subject	Treatment dose and duration	Brain region	Molecular mechanism and outcomes	References
1	*Panax ginseng*	PGE	CRS	2 h each day for 14 days	Adult male C57BL/6 mice	75, 150, 300 mg/kg (p.o.) for 14 days	Am	BDNF ↑; neuroinflammatory response ↓; Nrf2 signalling pathway↑; MAPKs and NF-κB pathways ↑	Choi et al. (2018)
2	*Panax ginseng*	PSG	LPS	0.5 mg/kg, (i.p.), single	Male ICR mice	0.25, 1 mg/kg (i.p.) for 7 days	HP	IL-6, TNF-α↓; SOD activation ↑; BDNF, TrkB, Sirt 1 ↑; IκB-α, NF-κB ↓	Wang et al. (2018)
3	*Panax ginseng C.A. Meyer*	GTS	LPS	0.8 mg/kg (i.p.), single	Male CD-1 mice	200 mg/kg (i.g.) for 7 days	HP	IL-1β, IL-6, TNF-α, IDO, CORT ↓	Kang et al. (2011)
4	*Panax ginseng*	Ginsenoside Rg1	LPS	5 μg (icv), single	Adult male Wistar rats	10, 30 mg/kg (i.p.) for 4 days	Cortex, HP	Deregulation of the hypothalamic–pituitary–adrenal axis ↓; microglia activation; pro-inflammatory mediators IL-6 ↓; peripheral corticosterone ↓	Zheng et al. (2014)
5	*Radix Ginseng*	Ginsenoside Rg1	CUMS	3 weeks	Male SD rats	20, 40 mg/kg (i.p.) for 3 weeks	PFC	IL-1β, NF-κB/NLRP3 pathway ↓	Zhang et al. (2019)
6	*Panax ginseng C.A. Meyer*	Ginsenoside Rg1	CUMS	5 weeks	Male Wistar rats	40 mg/kg, (i.p.) for 5 weeks	vmPFC	Iba-1, GFAP↑; p-CREB, BDNF, PSD-95, Synaptophysin↑; Bcl-2, Nrf2↑;IL-1β, IFN-γ, TNF-α↓; Neuronal apoptosis, Caspase-3, Caspase-9, (p-p38 MAPK),κB (NF-κB) p65 ↓	Fan et al. (2018)
7	*Panax ginseng C.A. Meyer*	Ginsenoside Rg1	LPS	LPS (3 μl, 1.67 mg/ml) was injected into the right cerebral ventricle, single	Male C57BL/6 mice	5, 10, 20 mg/kg (p.o.) for 3 days	PFC, HP	TNF-α, NO ↓; Iba-1, microglial activation ↓; NF-κB and MAPK pathway ↓	Hu et al. (2011)
8	*Panax ginseng*	Ginsenoside Rg1	CUMS	5 weeks	Male Wistar rats	40 mg/kg (i.p.) for 5 weeks	HP	Bcl-2 ↑;NOX, Bax, Caspase-3, Caspase-9 ↓; IL-1β, IFN-γ, TNF-α ↓	Li et al. (2020)
9	*Panax ginseng*	Ginsenoside Rg1	CRS	28 days	Male SD rats	20 mg/kg/days (i.g.)	HP	GAS5 ↓; microglial activation ↓; TNF-α, IL-1β, IL-6 ↓; SOCS3, NRF2, EZH2 ↑	Li et al. (2022)
10	*Red ginseng*	Ginsenoside Rg3	LPS	0.83 m/kg (i.p.), single	Male ICR mice	20, 40 mg/kg (i.g.)	HP	IL-6, TNF-α, Pro-inflammatory cytokines ↓	Kang et al. (2017)
11	*Panax ginseng C.A. Meyer*	Ginsenoside Rg3	LPS	3 mg/kg (i.p.), single	Male C57BL/6 mice	10, 20, 30 mg /kg (p.o.)	Cortex, DG, Hypothalamus	Microglia activation ↓; TNF-α, IL-1β, IL-6 ↓; iNOS, COX-2 ↓	Park et al. (2012)
12	*Panax ginseng C.A. Meyer*	Ginsenoside Rb1	CRS	21 days	Male ICR mice	10 mg/kg (i.p.) for 14 days	HP	BDNF, p-AKT/AKT ↑; IL-1β,TNF-α ↓	Guo et al. (2021)
13	*Panax ginseng*	Ginsenoside Rb1	CSDS	5 min for 28 days	Male C57BL/6 J mice	35, 70 mg/kg (p.o.)	HP	TNF-α, IL-18, IL-1, Iba1, NLRP3 ↓; Nrf2, HO-1, SIRT1 ↑	Jiang et al. (2022c)
14	*Panax ginseng*	Ginsenoside Rb1	CMS	8 weeks	Adult male C57BL/6 J mice	20 mg/kg/days (i.g.) for 4 weeks	Cortex, HP	Microglia activation ↓; TNF-α, IL-1β ↓; TGF-β, Arg-1, p-PPARγ ↑; neurogenesis ↑	Zhang et al. (2021)
15	*Panax ginseng Meyer*	Ginsenoside Rb1	LPS	1 mg/kg, single	Male ICR mice	10, 20 mg/kg (p.o.) for 11 days	HP	MAPK/NF-κB ↓; 5-HT level and 5-HT1A receptor ↑	Liang et al. (2022)
16	*Red ginseng*	Ginsenoside Rd	IS and EC	IS (12 h/days) for 2 days; EC (1 × 109 CFU/mouse/days, suspended in 0.2 mL saline) for 5 days	C57BL/6	5 mg/kg (p.o.), once a day for 5 days	HP	BDNF, BDNF/NeuN cell population ↑; CORT, NF-κB activation, TNF-α, IL-6, NF-κB/CD11c cell population ↓	Han et al. (2020)
17	*Red ginseng*	Ginsenoside Rh2	CRC	orthotopic implantation	Female NOD/SCID mice	0.2, 1, 5 mg/kg twice per week for 4 weeks	/	IL-6, IL-1,TNF-α ↓	Wang et al. (2016a, b)
18	*Panax ginseng Meyer*	Ginsenoside Rh2	Maternal Toxoplasma gondii (T. gondii) infection	pregnant	BALB/c mice	50, 100 mg/kg (i.g.)	PFC	Activation of microglia, IκB-α, p-NF-κB p65, neuroinflammation ↓	Xu et al. (2022)
19	*Panax ginseng*	Ginsenoside Rk1	LPS	0.83 mg/kg LPS (i.p.), single	Adult male ICR mice	5, 10, 20 mg/kg (i.g.) for 7 days	HP	SOD, BDNF, TrkB ↑; inflammatory factor, Sirt1, p-NF-κb/NF-κb,; p-IκB-α/IκB-α ↓	Li et al. (2020)
20	*Panax ginseng*	20(S)-Protopanaxadiol	CUMS	5 weeks	Male Sprague–Dawley rats	20, 40 mg/kg (i.p.) for 14 days	PFC, HP	CORT, proinflammatory cytokines (IL-6, IL-1β and TNF-α), microglial activation, 5-HT and NE↓; iNOS, COX2, caspase-9, caspase-3, Bax, Bcl-2, ac-p65↓	Jiang et al. (2020a, b)
21	*Panax notoginseng*	Notoginsenoside R1 (NGR1)	CUMS	21 days	Male Wistar rats	50, 100 mg/kg (p.o.) for 1 weeks	HP	PI3K/AKT/NF-κB ↓; TNF-α, IL-6, IL-1β ↓	Zhan et al. (2022)

5-HT, 5-hydroxytryptamine; Am, Amygdala; AKT, Protein kinase B; Arg-1, Arginase-1; Bcl-2, B cell lymphoma-2; BDNF, Brain-derived neurotrophic factor; CMS, Chronic mild stress; CORT, Corticosterone; COX-2, Cyclooxygenase-2; CRC, Colorectal carcinoma; CREB, cAMP-response element binding; CRS, Chronic restraint stress; CSDS, Chronic social defeat stress; CUMS, Chronic unpredictable mild stress; EC, Escherichia coli; EZH2, Enhancer of zeste homolog 2; GAS5, Growth arresting-specific 5; GFAP, Glial fibrillary acidic protein; GTS, Ginseng total saponins; HO-1, Heme oxygenase-1; HP, hippocampus; Iba-1, Ionized calcium binding adapter molecule-1; IDO, Indoleamine 2,3-dioxygenase; IFN-γ, Interferon-gamma; IL-1β, Interleukin-1beta; IL-6, Interleukin- 6; iNOS, Inducible nitric oxide synthase; IS, Immobilization stress; IκB-α, Inhibitor of κB-alpha; LPS, lipopolysaccharide; MAPK, Mitogen-activated protein kinase; NE, Norepinephrine; NF-κB, Nuclear factor kappa-B; NLRP3, NOD-like receptor thermal protein domain associated protein 3; NOX, NADPH oxidases; Nrf2, Nuclear factor-erythroid 2 p45-related factor 2; PFC, Prefrontal cortex; PGE, P. ginseng extract; PI3K, Phosphoinositide 3-kinase; PPAR, Peroxisome proliferator-activated receptor; PSD-95, Postsynaptic density protein-95; PSG, *Panax ginseng*; Sirt 1, Sirtuin type 1; SOCS3, Suppressors of cytokine signaling; SOD, Suppressed superoxide dismutase; TNF-α, Tumor necrosis factor-alpha; TrkB, Tropomyosin-related kinase B; vmPFC, Ventromedial prefrontal cortex

PPD reduced elevated serum levels of CORT and pro-inflammatory cytokines (IL-6, IL-1β, and TNF-α), as well as elevated levels of neurotransmitters (5-HT and NE) in hippocampal and PFC in CUMS mice (Jiang et al. 2020a, b). Besides, PPD-treated rats showed reduced hippocampus levels of iNOS, COX2, cleaved-caspase3, cleaved-caspase9, Bax, Bcl-2, and ac-p65, and increased levels of Sirt1 (Jiang et al. 2020a, b). Nottoginsenoside R1 (NGR1) exerts significant roles in anti-inflammatory, antioxidant, and anti-apoptotic activities (Zhan et al. 2022). Zhan et al. concluded that NGR1 alleviates depressive-like behavior by regulating the PI3K/Akt/NF-κB pathway (Zhan et al. 2022). Rg1 treatment also alleviates depression-like behaviors by reducing microglial activation in CRS rats (Li et al. 2022). In addition, a meta-analysis of clinical trials has shown that ginseng supplementation can reduce serum CRP/hsCRP levels in patients with elevated serum levels of this inflammatory marker, indicating that possible correlation with depression (Saboori et al. 2019). These findings suggest the potential effects of ginseng and ginsenosides against COVID-19-induced depression, especially given the role of inflammatory factors.

### Ginseng alleviates COVID-19 pandemic-caused depression by targeting inflammasomes

Currently, there are no known specific treatments that can inhibit SARS-CoV-2 infection and cure neurological symptoms such as depression caused by the infection. Due to the lack of definitive treatment options, natural remedies like ginseng and its derivatives are widely employed as immune boosters or health supplements to prevent SARS-CoV-2 infection and alleviate neurological manifestations, including depression, associated with COVID-19 (Shin and Cho 2023; Tian et al. 2022). Ginseng, along with its key active components such as ginsenosides and saponins, exhibits immunomodulatory properties and anti-inflammatory effects by modulating inflammasome activity (Jung and Lee 2022). SARS-CoV-2 can directly or indirectly affect the sensor nucleotide-binding oligomerization structural domain, leucine-rich repeat sequence, and NLRP3 of inflammasomes, ultimately leading to the assembly of NLRP3 inflammasome and the activation of inflammatory caspases, thereby inducing an inflammatory disruption of severe COVID-19 (Jung and Lee 2022). Related studies have shown that Korean Red Ginseng has an inhibitory effect on NLRP3 inflammasome vesicles and an ameliorative effect on a variety of NLRP3 inflammasome vesicle-mediated diseases (Han et al. 2017; Kim et al. 2014). Patients with chronic and persistent hyperactivation of NLRP3 inflammasome vesicles infected with SARS-CoV-2 have a fatal prognosis (Reyes et al. 2021). NLRP3 inflammasomes are significant pathogenic factors in metabolic, neurodegenerative, and psychiatric diseases (Shahzad et al. 2022). Regular consumption of KRG as a supplement inhibits excessive activation of NLRP3 inflammasome and may alleviate the progression of severe COVID-19 symptoms(Jung and Lee 2022). It has been reported that Ginsenosides Rb1 produces an antidepressant-like effect by regulation of SIRT1-NLRP3/Nrf2 pathways in chronic social defeat stress mice (Jiang et al. 2022b). In addition, Ginsenoside Rg1 attenuates depressive-like behavior by regulating the NF-κB/NLRP3 pathway in chronic unpredictable mild-stress rats (Zhang et al. 2019). Moreover, saponins from Panax japonicus alleviate HFD-induced depressive-like behaviors via inhibiting NLRP3 inflammasome (Wang et al. 2021a). Therefore, ginseng alleviates COVID-19 pandemic depression by regulating NLRP3 inflammasomes.

### Effects of ginseng on COVID-19 caused cognitive impairment

COVID-19 pneumonia leads to a cognitive dysfunction called "coronavirus fog", which may be associated with neuroglial dysregulation and neural circuit dysfunction. Hypoxia in selected brain regions may favor the ability of the virus to reproduce, and the integration of the viral genome in hypoxic brain regions leads to impaired metabolism of brain tissue capacity, which in turn leads to impaired energy supply, thus affecting cognitive function quite broadly (Stefano et al. 2021). Especially in the elderly, the severity of COVID-19 pneumonia, delirium, and chronic obstructive pulmonary disease (COPD) are risk factors for cognitive impairment. Patients with severe disease cases should be closely monitored for cognitive decline after COVID-19 infection (Liu et al. 2021c). A study has found that inflammatory factors including IL-2, YKL40, IL-4, IL-6, IL-10, sCD40L, TNF-α, IL-1Ra, interferon-gamma (IFN-γ), and CRP, are associated with cognitive impairment in COVID-19 patients (Zhou et al. 2020a). It has been reported that the ginsenosides Rg1 and Rf improve memory loss and cognitive dysfunction by regulating NF-κB, NLRP1, TLR3, and TLR4 signaling pathways, or interferon-gamma (IFN-γ) and active caspase-1 in an Alzheimer’s disease model (Du et al. 2018; Wu et al. 2022). KRG modulates anti-inflammatory activity via the NF-κB and BDNF pathways and exerts a memory-improving effect in a single chronic stress (SPS)-induced model of post-traumatic stress disorder (Lee et al. 2022). Ginsenoside Rd ameliorates cognitive impairment in a mouse model of chronic restraint stress (CRS) by attenuating oxidative stress and inflammation, and by upregulating the hippocampal BDNF-CREB signaling pathway (Wang et al. 2020). Panax notoginseng saponins attenuate CCL2-induced cognitive dysfunction in rats via anti-inflammatory and anti-apoptotic effects (Zhou et al. 2020b). Ginsenoside Rg1 inhibits neuroinflammation, protects neurons, and promotes neuroplasticity in brain regions associated with cognitive processing by regulating microglia and cytokines, resulting in anti-chemobrain effects that are associated with inhibition of neuroinflammation (Shi et al. 2019). In addition, a clinical study reported that Shenmai and Shenfu treatment, two ginseng-containing formulations, may enhance cognition via decreases in inflammatory factors (Zhang et al. 2018). As shown in Table 2, ginsenosides have positive effects in many different animal models of memory impairment with diverse bases. There is experimental evidence that ginseng extract can depress ACE activity in endothelial cells, which may contribute to improved cognitive performance in these models. In addition, the ginsenoside Re (GRe), but not the ginsenoside Rb1 (GRb1), significantly impaired the increasing expression of AT1 receptors in aged klotho-deficient mice (Nguyen et al. 2022). These results indicated that ginseng may improve COVID-19-caused cognitive impairment via inhibition of inflammation and ACE.

**Table 2:** Inflammation-mediated cognition-improvement effect of ginseng in different animal models

No.	Ginseng component	Active dose and treatment duration	Diseases	Animal species	Model preparation	Molecular mechanisms	References
1	Ginsenoside Rg1	–	AD	–	–	Regulating NF-κB, NLRP1, TLR3, and TLR4 signaling pathways	Ding et al. (2022)
2	Ginsenoside Rf	20 mg/kg，(i.p.) for 2 weeks	AD	Male C57Bl/6 mice	Intraventricular injection of beta-amyloid peptide	IFN-γ, active caspase-1 expression ↓; IL-13 expression ↑; Aβ clearance speed ↑	Du et al. (2018)
3	KRG	20, 50, 100 mg/kg, (i.p.) for 14 days	PTSD	Male Sprague-Dawley rats	SPS	Regulating NF-κB and BDNF pathway	Lee et al. (2022)
4	Ginsenoside Rd	10, 20 or 40 mg/kg, (p.o.) for 28 days	Cognitive impairment	Male C57BL/6J mice	CRS for 35 days	Oxidative stress and inflammation ↓; hippocampal BDNF-mediated CREB signaling pathway ↑	Wang et al. (2020)
5	PNS	50, 100, 200 mg/kg/days for 3 days	HAND	Sprague-Dawley (SD) male rats	CCL2 injection (5 ng of 1 ng/μl)	Inflammation and apoptosis effects ↓	Zhou et al. (2020a, b
6	Ginsenoside Rg1	5, 10 mg/kg/daysfor 3 weeks	Chemobrain	Female C57BL/6J	Three injections of docetaxel, adriamycin, and cyclophosphamide (DAC) in combination at a 2-day interval	Modulating microglia-mediated cytokines and the related upstream mediators	Shi et al. (2019)
7	Shenmai or Shenfu	2 ml (i.v.) every 8 h for 3 days	POCD	Aged Sprague-Dawly rats	Nnderwent splenectomy under general anesthesia	Inflammatory factor (IL-6, TNF-α) ↓; COR, ALD, ACTH ↓	Zhang et al. (2018)
8	Ginsenoside Rg1	200 mg/kg (i.p.) for 30 days	Cognitive impairment	Wistar male rats	LPS (500 μg/kg, i.p.), single	Prevented LPS-induced decrease in ACh levels and increase of AChE activity; Reverted the decrease of α7-nAChR protein expression in the PFC and HP	Jin et al. (2017)
9	WGOS	40, 80 mg/kg (i.p.) for 30 days	Cognitive impairment	Male ICR mice	SCO-induced model (3 mg/kg)	Pretreatment scopolamine-induced hyperexpression of proinflammatory cytokines IL-1β and IL-6 mRNA ↓; astrocyte activation in the HP ↓	Xu et al. (2016)
10	Ginsenoside Rg5	5, 10, 20 mg/kg (p.o.) for 28 days	AD	Wistar rats	STZ-induced model (3 mg/kg, i.c.v.)	Inflammatory cytokines TNF-α and IL-1β ↓; AChE activity ↓; Aβ deposition ↓; IGF-1, BDNF ↑	Chu et al. (2014)
11	Panax ginseng	50, 100, 200 mg/kg (p.o.) for 2 weeks	Brain injury-induced cognitive dysfunction	Adult male Wistar rats	Traumatic brain injury model	Neuroinflammation (TNF-α and IL-6), AchE levels ↓; microglia activation ↓	Kumar et al. (2014)

Ach, Acetylcholine; AchE, Acetylcholinesterase; ACTH, Adrenocorticotropic hormone; AD, Alzheimer's disease; ALD, Aldosterone; Aβ, Amyloid-β peptides; BDNF, Brain-derived neurotrophic factor; CCL2, Chemokine CC motif ligand 2; COR, Cortisol; CREB, cAMP-response element binding; CRS, Chronic restraint stress; HAND, HIV-associated neurocognitive disorders; HP, hippocampus; IFN-γ, Interferon-gamma; IGF-1, Insulin-like growth factors-1; IL-13, Interleukin-13; IL-1β, Interleukin-1beta; IL-6, Interleukin-6; KRG, Korean Red Ginseng; LPS, lipopolysaccharide; NF-κB, Nuclear factor kappa-B; NLRP1, Nod-like receptor protein 1; PFC, Prefrontal cortex; PNS, Panax notoginseng saponins; POCD, Postoperative cognitive dysfunction; PTSD, Post-traumatic stress disorder; SCO, Scopolamine; SPS, Single prolonged stress; STZ, Streptozotocin; TLR3, Toll-like receptors 3; TLR4, Toll-like receptors 4; TNF-α, Tumor necrosis factor-alpha; WGOS, Water-soluble ginseng oligosaccharides.

### Anti-fatigue effect of the ginseng and ginsenosides

**Fig. 2:**
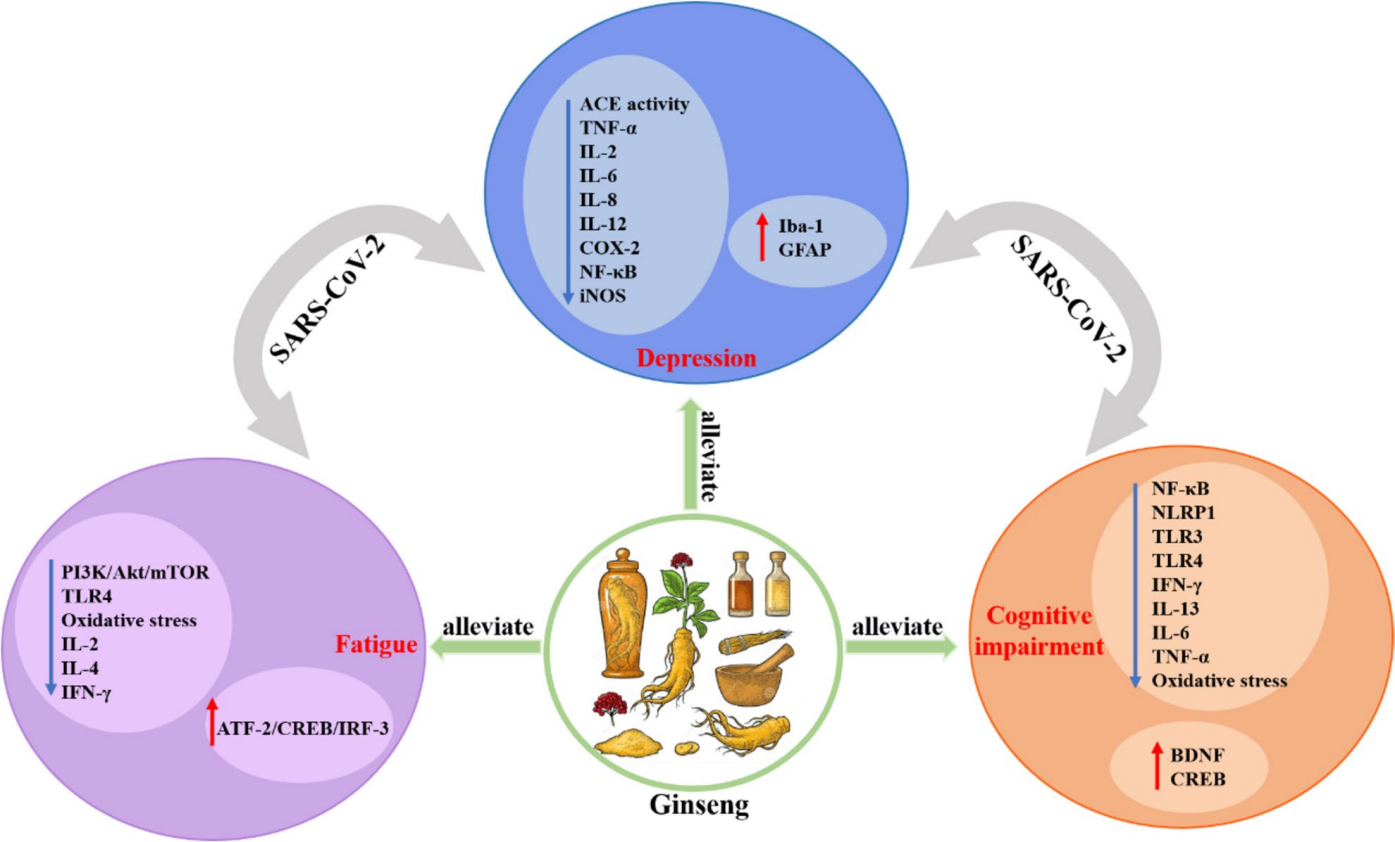
Ginseng may alleviate depression, cognitive impairment, and fatigue caused by SARS-CoV-2 through the different inflammatory pathways, molecules as well as relevant receptors. SARS-CoV-2, Severe Acute Respiratory Syndrome Coronavirus 2; ACE, angiotensin-converting enzyme 2; TNF-α, tumor necrosis factor-α; IL-2, Interleukin-2; IL-4, Interleukin-4; IL-6, Interleukin-6; IL-8, Interleukin-8; IL-12, Interleukin-12; IL-13, Interleukin-13; COX-2, cyclooxygenase 2; NF-κB, nuclear factor kappa-light chain-activated B-cell enhancer; iNOS, Inducible nitric oxide synthase; Iba-1, Ionized calcium binding adapter molecule-1; GFAP, glial fibrillary acidic protein; PI3K, Phosphoinositide 3-kinase; AKT, protein kinase B; mTOR, mammalian or mechanistic target of rapamycin; TLR3, Toll-like receptor 3; TLR4, Toll-like receptor 4; IFN-γ, interferon-gamma; ATF2, Activating transcription factor 2; CREB, cAMP-responsive element-binding protein; IRF3, Interferon regulatory factor 3; NLRP1, Nod-like receptor protein 1; BDNF, brain-derived neurotrophic factor

Fatigue is a feeling of exhaustion, a symptom or co-morbidity of many neuropsychiatric disorders. Of survivors of COVID-19 pneumonia, 72.8% showed extreme fatigue symptoms, similar to those following other severe acute respiratory syndrome infections (Kamal et al. 2021; El Sayed et al. 2021). Chronic fatigue is a frequent symptom of many neurological disorders. It manifests as peripheral fatigue dominated by the inability to maintain muscle contractility and central fatigue affecting central, peripheral, and autonomic nervous system functions. In this type of extreme fatigue, the metabolism and activity of neurotransmitters are altered by the release of inflammatory mediators from activated intrinsic immune cells in the peripheral and central nervous systems (Dantzer et al. 2014). This relationship has been demonstrated in several animal models. A recent study has shown that ginseng may be a potential exercise energizer, triggering metabolic adaptation to energy consumption by activating the PI3K/Akt/mTOR signaling pathway, and that short-term EEP (ethanol extraction of *P. ginseng* roots) supplementation may significantly improve exercise capacity (Zhang et al. 2023). Moreover, ginseng has excellent anti-fatigue properties and may have future potential as an herbal medicine for anti-fatigue treatment (Zhang et al. 2023; Bach et al. 2016). Related studies have also shown that small molecule oligopeptide, *Panax ginseng* polysaccharide isolated from *Panax ginseng* reduced immobility time in the forced swimming test and produced anti-fatigue effects by inhibiting oxidative stress and improving mitochondrial function in skeletal muscle (Bao et al. 2016; Li et al. 2018). Vina-ginsenoside R2 and alongside R2 and their metabolites isolated from Panax vietnamensis inhibit inflammation by inhibiting the binding of LPS to TLR4 on macrophages, a mechanism that may also be related to the anti-fatigue and anti-inflammatory effects of ginseng (Jeong et al. 2015). It also has been reported that Korean red ginseng water extract produces anti-inflammatory activity via the ATF-2/CREB/IRF-3 pathway (Yang et al. 2014). Kai Xin San (KXS), a herbal formulation consisting of ginseng (*Panax ginseng*), hoelen (*Wolfiporia cocos*), polygala (*Polygala tenuifolia*) and *Acorus gramineus*, produces anti-fatigue effects by modulating interleukin-2 (IL-2) and interleukin-4 (IL-4) in a model of chronic fatigue syndrome induced by forced wheel running (Cao et al. 2012). All of the above evidence suggests that ginseng may alleviate depression-like symptoms, cognitive dysfunction and fatigue after SARS-CoV-2 infection through an inflammatory pathway (Jiang et al. 2022a; Jeon and Kim 2016; Zhou et al. 2020a, b; Jeong et al. 2015; Wang et al. 2021b) (Fig. 2).

## Conclusion

This review concludes that ginseng may be effective in improving depression, fatigue, and cognitive deficits in some psychiatric and neurological conditions. Based on the known mechanisms of COVID-19 post-infection syndrome, ginseng, ginseng extracts, or ingredients may be especially effective in treating the neuropsychiatric outcomes of this condition. The common mechanisms involved act to suppress inflammation by reducing the levels of IL-13, IL-4, IL-5, and IL-8, and through actions on the TNF-α/NF-kB pathway. In addition, RAAS is also involved in the anti-depression, anti-fatigue, and cognition-improving effects of ginseng, and may thus be useful in the treatment of post-COVID-19 neuropsychiatric syndrome. Relevant clinical trials are needed to test this hypothesis in the future to further validate the clinical application of ginseng against post-COVID-19 neuropsychiatric syndrome, as well as other neuropsychiatric diseases that may share common underlying inflammatory bases.

## Future directions

Until now, there are no effective pharmaceuticals for COVID-19, so we can only target the complications linked to COVID-19 to reduce the mortality of infected patients. Despite the clinical efficacy of natural products on COVID-19 remains to be investigated, we suggest ginseng as a promising candidate herbal medicine for supplementation in COVID-19 patients, which could potentially prevent inflammation and COVID-19-related depression, fatigue, and cognitive impairment. Of course, this will require further extensive clinical studies to evaluate. In the future, research will focus on how different types of ginseng and its extracts could be selected by COVID-19 patients with different body types to improve psychiatric disorders caused by COVID-19. In addition, we will delve deeper into understanding the distinctions between different types of ginseng and their varying effects on inflammatory factors. This will ultimately contribute to providing more targeted and personalized treatment options for COVID-19 patients.

## Data Availability

Not applicable.
